# Unstable Angina as a Component of Primary Composite Endpoints in Clinical Cardiovascular Trials: Pros and Cons

**DOI:** 10.1159/000524948

**Published:** 2022-05-10

**Authors:** Anna Meta Dyrvig Kristensen, Manan Pareek, Kristian Hay Kragholm, Thomas Steen Gyldenstierne Sehested, Michael Hecht Olsen, Eva Bossano Prescott

**Affiliations:** ^a^Department of Cardiology, Copenhagen University Hospital − Bispebjerg and Frederiksberg, Copenhagen, Denmark; ^b^Department of Cardiology, Copenhagen University Hospital − Herlev and Gentofte, Copenhagen, Denmark; ^c^Department of Cardiology, Aalborg University Hospital, Aalborg, Denmark; ^d^Department of Cardiology, Zealand University Hospital Roskilde, Roskilde, Denmark; ^e^Division of Cardiology, Department of Internal Medicine, Holbæk Hospital, Holbæk, Denmark; ^f^Department of Regional Health Research, University of Southern Denmark, Odense, Denmark

**Keywords:** Acute coronary syndromes, Angina, Unstable angina, Clinical trials, Composite endpoint

## Abstract

**Background:**

Unstable angina (UA) is a component of acute coronary syndrome that is only occasionally included in primary composite endpoints in clinical cardiovascular trials. The aim of this paper is to elucidate the potential benefits and disadvantages of including UA in such contexts.

**Summary:**

UA comprises <10% of patients with acute coronary syndromes in contemporary settings. Based on the pathophysiological similarities, it is ideal as a part of a composite endpoint along with myocardial infarction (MI). By adding UA as a component of a primary composite endpoint, the number of events and feasibility of the trial should increase, thus decreasing its size and cost. Furthermore, UA has both economic and quality of life implications on a societal and an individual level. However, there are important challenges associated with the use of UA as an endpoint. With the introduction of high-sensitivity troponins, the number of individuals diagnosed with UA has decreased to rather low levels, with a reciprocal increase in the number of MI. In addition, UA is particularly challenging to define given the subjective assessment of the index symptoms, rendering a high risk of bias. To minimize bias, strict criteria are warranted, and events should be adjudicated by a blinded endpoint adjudication committee.

**Key Messages:**

UA should only be chosen as a component of a primary composite endpoint in cardiovascular trials after thoroughly evaluating the pros and cons. If it is chosen to include UA, appropriate precautions should be taken to minimize possible bias.

## Introduction

Unstable angina (UA), non-ST-segment elevation myocardial infarction (NSTEMI), and ST-segment elevation myocardial infarction (STEMI) comprise the three main presentations of acute coronary syndromes (ACS) [1]. Traditionally, myocardial infarction (MI), alongside death from cardiovascular causes and stroke, have been the preferred components of endpoints in cardiovascular clinical trials [2]. However, several large trials have also included UA as a component of their primary composite endpoint. Nevertheless, there is some reluctance to its use. The purpose of this paper is to describe the potential challenges and opportunities associated with using UA as a component of a primary composite endpoint.

## Definition and Pathophysiology of UA

A diagnosis of UA is based on symptoms suggestive of myocardial ischemia and the absence of acute myocardial injury or necrosis (i.e., no dynamic elevation of cardiac troponin) [3, 4, 5]. UA should be suspected when the patient presents with resting angina for >20 min, new-onset angina, or crescendo angina, defined as a change from previous episodes in severity, intensity, or duration, which may occur with minimal physical exertion [3]. The ECG may show ST-segment depression, transient (≤20 min) ST-segment elevation, T-wave inversion, or may be normal.

**Fig. 1:**
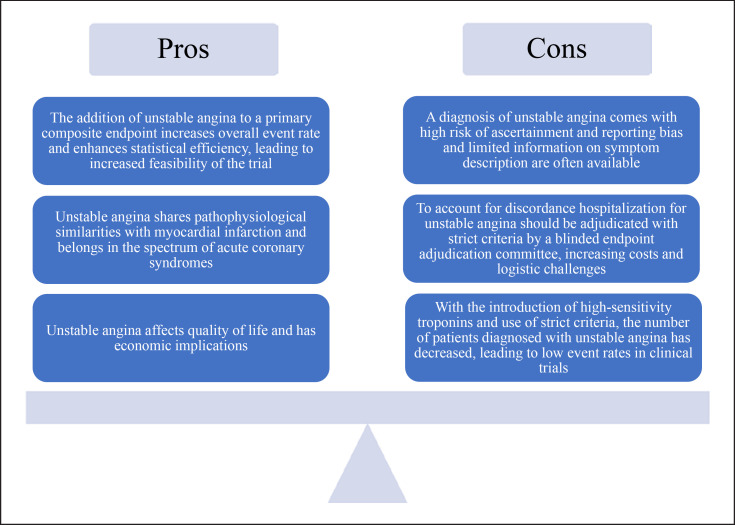
Pros and cons of using UA as an endpoint in cardiovascular clinical trials.

The major international cardiovascular societies categorize UA and NSTEMI together using the term NSTE-ACS [3, 5]. ACS is mainly attributed to plaque rupture, resulting in thrombosis and complete or incomplete occlusion of a coronary artery [6]. An additional cause of ACS includes plaque erosion. Newer studies suggest that plaque erosion actually comprises 30–40% of ACS [7], a change in epidemiology largely believed to be a result of better risk factor control [8]. Generally, plaque erosion is associated with NSTE-ACS [7]. The close pathophysiological link between UA and MI serves as one argument for using UA as a clinical endpoint in line with MI. A summary of the pros and cons is presented in Figure 1. Practical guidance when considering UA as an endpoint is presented in Table 1 and further discussed below.

## Epidemiology of UA

During the last decades, better risk factor control, evolving MI definitions with widespread use of high-sensitivity troponins, and reperfusion strategies have changed the epidemiology and prognosis of patients diagnosed with UA. The 2002 Euro Heart Survey of ACS based on data from 25 countries reported that UA comprised 42% of patients with an ACS [9]. Conversely, in the era of high-sensitivity troponins, a Danish cohort study found that less than 10% of patients with ACS were diagnosed with UA [10]. Similar results have been reported in other contemporary studies [11, 12]. Interestingly, patients diagnosed with UA generally appear to be younger than those with NSTEMI but present with a higher prevalence of most cardiovascular risk factors and more advanced coronary artery disease (CAD) [11, 13, 14].

Patients with UA also appear to derive less benefit from intensified antiplatelet therapy and an invasive strategy within 72 h, although invasive coronary angiography is still recommended in patients with a high likelihood of UA [3]. A contemporary Swedish registry-based study found that ∼88% of patients diagnosed with UA underwent invasive coronary angiography and ∼75% underwent revascularization [13]. In contrast, a contemporary German study found that coronary angiography was performed in ∼72%, but only ∼29% underwent revascularization [12]. The discrepant findings may reflect differences in the definition of UA as well as differing practices related to referral to invasive assessment, which may again complicate the use of UA as a component of a primary composite endpoint.

## Prognosis of Patients with UA

Generally, the risks of subsequent death and cardiovascular events are lower in the UA setting compared with NSTEMI [3, 13]. For example, one report based on two independent prospective multicenter studies (*n* = 8,992) found that the rate of incident nonfatal MI was similar in patients with UA and NSTEMI, but all-cause mortality was considerably lower in those with UA [11]. Another study comparing outcomes of percutaneous coronary intervention-treated patients with UA, stable angina, and NSTEMI (*n* = 7,187, 38% with a diagnosis of UA) also reported substantially better outcomes in patients with UA compared with NSTEMI [14]. Nevertheless, after adjustment for baseline differences, event risks were found to be comparable. According to a registry-based study including 3,204 patients with UA, 6.3% of the patients died within the first year after the diagnosis [13].

## UA in the Era of High-Sensitivity Troponin Assays

High-sensitivity cardiac troponin assays have improved sensitivity and precision, enabling more rapid and accurate diagnosis in patients with suspected MI [15, 16]. The assays have superior precision at the 99th percentile and enable these to be more accurately defined. Accordingly, the introduction of high-sensitivity cardiac troponins has altered the epidemiology of UA and MI [17]. Considering unselected patients presenting with suspected NSTE-ACS, the use of such assays has enabled the identification of smaller MI including type 2 MI and the incidence of MI has increased (4% absolute and 20% relative increase) with a reciprocal decrease in UA [18]. It is notable however, that the general incidence of MI is declining [19]. With this decrease in the incidence of UA, it has been argued that UA no longer belongs within the clinical spectrum of ACS but could be viewed as a subgroup of severe stable CAD, serving as an argument for omitting UA as a component of a primary composite endpoint [11, 20, 21]. Conversely, a contemporary study found that the risk of death in UA patients was higher than in stable angina (10.5% of UA patients died within the first 3 years vs. 7.5% in patients with stable angina) [14].

Serial troponin testing with exclusion of a significant rise or fall in its concentration is key as some patients with UA may present with chronically elevated high-sensitivity cardiac troponin levels, e.g., patients with known chronic kidney disease, chronic coronary syndrome, or heart failure. Still, it can be difficult to distinguish UA from NSTEMI, e.g., in very late presenters of MI, where the troponin release curve has flattened. As opposed to the aforementioned position on grouping UA as a subgroup of stable CAD, other studies have stated that UA and NSTEMI should be grouped together and patients should be managed depending on their cardiovascular risk factors instead of the diagnosis [12, 13]. This position supports the use of UA as an endpoint in clinical cardiovascular trials.

## Composite Endpoints in Cardiovascular Clinical Trials

The ideal endpoint of a clinical trial is based on several considerations. It should be well-defined, easy to evaluate, clinically relevant to the intervention and the patient, and have a low risk of ascertainment and reporting bias. In addition, it should occur at a suitable frequency within a limited time frame for a trial to be feasible. All-cause mortality has fulfilled all these criteria. However, a declining mortality rate in the general population, including a declining proportion of mortality caused by cardiovascular disease, makes all-cause mortality less sensitive to interventions that primarily exert their beneficial effects through cardiovascular outcomes [22, 23]. Improved population health and novel treatments have also reduced cardiovascular event rates. Consequently, a single-component primary endpoint is likely to result in an unreasonably long follow-up period and/or large sample size. Therefore, composite endpoints are almost exclusively used in contemporary trials. A composite endpoint increases the total number of events, statistical power, and precision. Such endpoints are particularly valuable if the intervention has a similar beneficial effect on outcomes of equal importance, and if they share a common pathophysiological mechanism such as MI and UA. Nevertheless, individual tailoring of composite endpoints may lead to differing results and conclusions and potentially hamper proper comparisons of trials.

The differences in the event rate of each component of a composite endpoint must be as small as possible since the overall effect of an intervention will largely be determined by the dominant event [24, 25]. Several nonfatal endpoints like MI, UA, and coronary revascularization have a higher incidence (and thus by default occur earlier) than more fatal endpoints as death or resuscitated cardiac arrest. Both the incidence and severity of the components of the combined endpoints need consideration. Methods have been proposed to weight the components of a composite endpoint to counter these issues [26, 27]. Attempts to quantify weights have been based on expert judgement (i.e., clinical-investigator Delphi panels), preference methods (decisions made by individuals when confronted with different scenarios) and disability-adjusted life years (DALY) lost [28]. However, the process of assigning weights is not standardized in randomized clinical trials, can be time consuming, and may be costly.

A commonly used composite endpoint in cardiovascular trials is major adverse cardiovascular events (MACE). Although it has not been defined strictly, it usually comprises a composite of death from cardiovascular causes, MI, and stroke [29]. Indeed, in 2008, the US Food and Drug Administration (FDA) decided to recommend assessment of the cardiovascular safety profile of antidiabetic agents using this three-point MACE but stated that hospitalization for ACS (UA), urgent revascularization, and possibly other endpoints could be added [30]. An example of a trial adding UA and urgent revascularization to the traditional 3-point MACE for its primary composite endpoint was the *Thrombin Receptor Antagonist for Clinical Event Reduction in Acute Coronary Syndrome* (TRACER) trial [31]. The key secondary endpoint of the trial was the traditional 3-point MACE, consisting of a composite of death from cardiovascular causes, MI, or stroke. Curiously, it was found that the occurrence of the primary endpoint did not reach significance, whereas the secondary did.

## The Health Care Burden of UA

As seen in Figure 1, one argument for expanding a composite endpoint with UA would be to gain a broader perspective of the symptom burden and the consequences for the individual and society. The economic burden of UA is almost as high as that of MI during the first year after diagnosis [32]. Additionally, a study found high hospitalization rates for both UA (∼89%) and NSTEMI (∼99%) [12]. On the patient level, UA negatively affects quality of life [33, 34].

## UA in Clinical Trials

An important caveat of using UA as an endpoint in cardiovascular trials is the extent of ascertainment and reporting bias, particularly with respect to symptom description. The clinical distinction between UA, NSTEMI, and noncardiac chest pain can be challenging, even when applying current guideline recommendations. Limited information on symptom description and duration of ischemic symptoms may lead to refuted events due to a lack of event-specific data [13]. Compared with MI, clinical subjectivity plays a greater role in its ascertainment, and interpretations may differ markedly between clinicians. A registry-based study found that the proportions of UA among those with NSTE-ACS ranged from 4% to 22% in the 49 different participating hospitals, suggesting variations in the local perception of UA [13]. Therefore, it is essential that cardiovascular trials have a clear, operational definition of UA with strict and objective criteria.

In 2017, the Standardized Data Collection for Cardiovascular Trials Initiative established by the FDA proposed definitions for cardiovascular endpoints including hospitalization for UA to simplify the design and conduct of clinical trials and to enhance the ability to aggregate and compare data from multiple trials [35]. The full criteria for hospitalization for UA are presented in Table 2. In brief, the criteria consist of 4 elements: (1) ischemic symptoms with a duration >10 min at rest or in an accelerating pattern; (2) prompting an unscheduled hospitalization within 24 hours of the most recent symptoms; (3) absence of cardiac biomarker elevation; and (4) one of the following: ECG changes, evidence of inducible myocardial ischemia, angiographic evidence of CAD believed to be responsible for the ischemic symptoms or signs, or need for a coronary revascularization procedure.

An example of a trial using these criteria is the *Reduction of Cardiovascular Events with Icosapent Ethyl-Intervention Trial* (REDUCE-IT), which randomized statin-treated patients with elevated triglycerides to icosapent ethyl or placebo [36]. The composite endpoint was cardiovascular death, MI, stroke, coronary revascularization, or UA. When comparing the concordance between investigator-reported and adjudicated primary endpoints, it was ∼100% for death and coronary revascularization, 80–90% for MI and stroke, but only 53% for UA [37]. Investigator-reported events exceeded adjudicated rates of UA (283 vs. 108), often at the expense of underreporting MI. Other studies have reached the same conclusions. One study compared local interpretation and blinded evaluation of the admission ECG in patients with UA and non-Q-wave MI and found considerable differences [38]. Another study evaluated reported and adjudicated events in *the Stabilization of Atherosclerotic Plaque by Initiation of Darapladib Therapy* (STABILITY) trial [39]. It was found that 25.4% of the 1,407 reported UA events were classified as such by the adjudication committee, primarily due to crossover between MI and UA. Indeed, the risk of misinterpretation and heterogeneity with UA is particularly high in large, multinational randomized controlled trials in which endpoints are interpreted by several local investigators from different hospitals. The large degree of subjectivity associated with this diagnosis may lead to variation in adjudication. One way of maximizing concordance for a complex endpoint like UA would be to exclusively use a blinded endpoint adjudication committee. However, the use of a clinical endpoint adjudication committee increases the costs and logistic challenges of clinical trials. Recent evidence has challenged the unquestioned role of an endpoint adjudication committee with the use of other components of a primary endpoint than UA [40, 41, 42]. Consequently, the choice of UA as a primary endpoint might not be favorable, as the use of UA leads to an ubiquitous need for an adjudication committee.

## Clinical Trials Using Hospitalization for UA as a Component of the Primary Endpoint: The ISCHEMIA Trial Example

Several large trials have used UA as a component of a primary composite endpoint. Notable examples are seen in Table 3. *The International Study of Comparative Health Effectiveness with Medical and Invasive Approaches* (ISCHEMIA) is an example of a trial expanding the primary endpoint by including UA in an attempt to increase feasibility [43]. Interestingly, the original grant application had described a five-component primary endpoint consisting of cardiovascular death, nonfatal MI, resuscitated cardiac arrest, or hospitalization for UA or heart failure. Subsequently, the steering committee was allowed to alter this endpoint to cardiovascular death or MI, but with a contingency plan to switch back to the original endpoint to retain power in case of a lower-than-expected event rate [51]. Indeed, it is not uncommon for clinical trials to have lower than anticipated event rates. The specific reasons for the inclusion of hospitalization for UA were clinical relevance, quality of life and economic implications, and potential benefits of revascularization. A strict definition of UA resembling the one proposed by the Standardized Data Collection for Cardiovascular Trials Initiative was employed. However, despite the fact that high-sensitivity troponins were not systematically used by all trial sites, relatively few UA events were reported (48 UA events vs. 326 events with nonprocedural MI). Several trials using UA as a component of the primary endpoint have been faced with similar issues as presented in Table 3.

## Clinical Trials Using Urgent Revascularization as a Component of the Primary Endpoint: The FAME-2 Example

An alternative approach might be to use revascularization, as UA is closely linked to urgent revascularization. As an endpoint, revascularization is clinically feasible and pathophysiologically relevant. It is easy to evaluate, has a higher event rate than UA, and the use of an adjudication committee may not be as essential. Examples of clinical trials using revascularization as a component of a primary endpoint are presented in Table 4. *The Fractional Flow Reserve-Guided PCI* versus *Medical Therapy in Stable Coronary Disease* (FAME-2) trial included unplanned hospitalization leading to urgent revascularization as part of the primary composite endpoint consisting otherwise of death from any cause or nonfatal MI [57]. This adjudicated endpoint was counted only if patients were hospitalized unexpectedly because of persisting or increasing complaints of chest pain (with or without ST-T changes) and underwent a revascularization procedure during the same hospitalization. This definition has substantial similarities to the definition of UA as described previously, but may be more objective. The results of the trial at both 7 months and 5 years were driven mainly by urgent revascularization [58, 59]. The definition of urgent revascularization as well as the fact that it resulted in the premature termination of FAME-2 has been questioned [60]. Furthermore, unplanned and planned revascularization as endpoints in clinical trials may face challenges with respect to subjectivity. Indeed, the decision on revascularization as well as whether to classify it as planned or unplanned are influenced by many factors such as local practice, available resources, and geographical location. Especially in nonblinded trials, there is concern for selection bias as investigators may be more willing to recommend revascularization for patients in one of the treatment groups rather than managing the symptoms noninvasively.

## UA as a Registry-Based Endpoint

Recently, there has been an increasing interest in randomized controlled trials with registry-based follow-up as they are more feasible, cheaper, and allow for longer-term follow-up [61]. The diagnosis of UA in registries is more likely to be independent of the trial and possible bias balanced in the intervention and control group. A systematic review of the validity of acute cardiovascular outcome diagnoses recorded in European electronic health records found that the positive predictive value (PPV) of UA varied considerably [62]. PPV as low as 20–30% [63, 64] to as high as 88% have been reported [65]. Conversely, a higher PPV has consistently been reported for MI, at >90% [62]. Because of considerable between-study heterogeneity, the use of UA as a registry-based endpoint in clinical trials should be done very cautiously and after proper validation. One possible way to increase specificity could be to define UA based on hospitalization and urgent invasive coronary angiography or revascularization.

## Conclusions and Recommendations

This review summarizes the challenges and opportunities associated with using UA as a component of a primary composite endpoint in cardiovascular clinical trials. Arguments favoring the use of UA rely on its pathophysiological similarities to MI, the ability to enhance statistical power, the impact on quality of life, and the health care burden. However, widespread use of high-sensitivity troponins has reduced the incidence of UA. UA may also be subject to ascertainment bias, reporting bias, and discordance between clinicians. Therefore, we recommend that UA events should be adjudicated with strict criteria by a blinded endpoint adjudication committee, or alternatively be defined in combination with revascularization, resulting in a limited number of events. If UA is considered as an endpoint in clinical trials, we recommend evaluating our list of pros and cons in Figure 1 and the practical guidance and recommendations in Table 1. The use of UA as a component of a primary composite endpoint should be done with caution and after thorough consideration.

## Conflict of Interest Statement

Dr. Manan Pareek declares the following disclosures (all unrelated to the content of the present manuscript): Advisory Board − AstraZeneca, Janssen-Cilag; Speaker's Fee − AstraZeneca, Bayer, Boehringer Ingelheim, Janssen-Cilag. The other authors have no conflicts of interest to declare.

## Funding Sources

There are no sources of funding.

## Author Contributions

Anna Meta Dyrvig Kristensen, Manan Pareek, and Eva Irene Bossano Prescott drafted the manuscript. Anna Meta Dyrvig Kristensen, Manan Pareek, Eva Irene Bossano Prescott, Kristian Hay Kragholm, Thomas Steen Gyldenstierne Sehested, and Michael Hecht Olsen revised the manuscript for important intellectual content.

## Figures and Tables

**Table 1:** Practical guidance when considering UA as an endpoint in randomized trials

Points to evaluate before choosing UA as a part of a composite endpoint:
Is the use of UA as an endpoint necessary to ensure feasibility of the trial?
Is UA clinically relevant for the treatment investigated?
Has the incidence of UA been considered?
Should weighted outcomes be considered?
Is it possible to use an alternative endpoint with less bias?
Have the pros and cons of using UA as an endpoint been carefully considered?

If UA is chosen as a part of a composite endpoint, we recommend the following:
Use of strict and objective criteria to minimize possible bias.
These criteria should be easy to use and evaluate. The possibility to aggregate and compare data from multiple trials should be present. We recommend the use of the definition proposed in 2017 by the Standardized Data Collection for Cardiovascular Trials Initiative established by FDA.
Use of a clinical endpoint adjudication committee to streamline the process of evaluating events.
The use of UA as a pragmatic registry-based endpoint in clinical trials should be done very cautiously and after proper validation. One possible way to increase specificity could be to define UA based on urgent hospitalization and unplanned revascularization.

**Table 2:** Criteria for adjudication of UA hospitalization defined by the Standardized Data Collection for Cardiovascular Trials Initiative and the US Food and Drug Administration [30] Criteria for adjudication of hospitalization for UA

UA requiring hospitalization is defined as
1. Ischemic discomfort (angina, or symptoms thought to be equivalent) ≥10 min in duration occurring at rest or in an accelerating pattern with frequent episodes associated with progressively decreased exercise capacity.
*AND*
2. Prompting an unscheduled hospitalization within 24 hours of the most recent symptoms. Hospitalization is defined as an admission to an inpatient unit or a visit to an emergency department that results in at least a 24-hours stay (or a change in calendar date if the hospital admission or discharge times are not available).
*AND*
3. At least one of the following: a. New or worsening ST- or T-wave changes on resting ECG (in the absence of confounders, such as LBBB or LVH)
Transient ST-elevation (duration <20 minutes)
New ST-elevation at the J-point in two contiguous leads with the cut-points:
≥0.1 mV in all leads other than leads V2–V3 where the following cut-points apply:
≥0.2 mV in men ≥40 years (≥0.25 mV in men <40 years) or ≥0.15 mV in women.
ST-depression and T-wave changes
New horizontal or down-sloping ST depression ≥0.05 mV in two contiguous leads and/or new T-wave inversion ≥0.3 mV in two contiguous leads with prominent R-wave or R/S-ratio >1.
b. Definite evidence of inducible myocardial ischemia as demonstrated by: An early positive exercise stress test, defined as ST-elevation or ≥2 mm ST-depression prior to 5 METs
OR
Stress echocardiography (reversible wall motion abnormality) OR
Myocardial scintigraphy (reversible perfusion defect), OR
MRI (myocardial perfusion deficit under pharmacologic stress). and believed to be responsible for the myocardial ischemic symptoms/signs.
c. Angiographic evidence of new or worse ≥70% lesion (≥50% for left main lesion) and/or thrombus in an epicardial coronary artery that is believed to be responsible for the myocardial ischemic symptoms/signs.
d. Need for coronary revascularization procedure (PCI or CABG) for the presumed culprit lesion(s), as defined in 3c. This criterion would be fulfilled if revascularization was undertaken during the unscheduled hospitalization, or subsequent to transfer to another institution without interceding home discharge.
*AND*
4. Negative cardiac biomarkers and no evidence of acute MI

CABG, coronary artery bypass grafting; LBBB, left bundle branch block; LVH, left ventricular hypertrophy; METs, metabolic equivalents; MI, myocardial infarction; MRI, magnetic resonance imaging; PCI, percutaneous coronary intervention.

**Table 3:** Examples of clinical trials using UA as an endpoint

Trial name	Key inclusion criteria and sample size, *n*	Intervention versus control	Primary endpoint	Primary results	Definition of UA and use of an adjudication committee	Events, *n*
ISCHEMIA [43]	Patients with stable coronary disease after clinically indicated stress testing showed moderate or severe reversible ischemia on imaging tests or severe ischemia on exercise tests without imaging (*n* = 5,179)	Initial invasive versus conservative medical therapy	A composite of CV death, Ml, resuscitated cardiac arrest, hospitalization for UA, or heart failure	Intervention 5.3% versus control 3.4% (difference 1.9 percentage points; 95% CI: 0.8–3.0)	Prolonged ischemic symptoms at rest (usually ≥10 min in duration), or accelerating pattern of chest pain that occurs with a lower activity threshold considered to be myocardial ischemia upon final diagnosis resulting in an unscheduled visit to a healthcare facility resulting in an overnight stay generally within 24 h of the most recent symptoms, cardiac biomarkers not meeting Ml criteria, and at least one of the following: New or worsening ST or T wave changes on resting ECG. ECG criteria: ST segment shifts and T-wave changes: New horizontal or down-sloping ST depression >0.05 mV in 2 contiguous leads; and/or T inversion >0.1 mV in 2 contiguous leads, or new ST segment elevation >0.1 mV in 2 contiguous leads. The ST-T wave criteria only apply in the absence of findings that would preclude ECG analysis such as LBBB, LVH with repolarization abnormalities, preexcitation and pacemakers. Angiographic evidence of a ruptured/ulcerated plaque, or thrombus in an epicardial coronary artery believed to be responsible for the ischemic symptoms/signs. Use of an adjudication committee: yes	UA: *n =* 48 Total: *n =* 639 UA/total: 7.5%

REDUCE-IT [36]	Patients with established CV disease or diabetes and other risk factors, in addition to elevated triglyceride levels (*n =* 8,179)	Icosapent ethyl 2 g twice daily versus placebo	A composite of CV death, nonfatal Ml, nonfatal stroke coronary revascularization, or UA	Intervention 17.2% versus control 22.0% (HR 0.75; 95% CI: 0.68–0.83; *p* < 0.001)	The criteria defined by the standardized data collection for cardiovascular trials Initiative and the US Food and Drug Administration (see Table 2). Use of an adjudication committee: yes	UA: *n =* 265 Total: *n=* 1,606 UA/total: 16.5%

ODYSSEY OUTCOMES [44]	Patients with ACS 1–12 months earlier, elevated triglyceride levels and receiving statin therapy (*n =* 18,924)	Alirocumab every 2 weeks versus placebo	A composite of death from coronary heart disease, nonfatal Ml, fatal or nonfatal ischemic stroke, or UA requiring hospitalization	Intervention 9.5% versus control 11.1% (HR 0.85; 95% CI: 0.78–0.93; *p <* 0.001)	A diagnosis of UA (*n*ew ACS event without elevations in cardiac biomarkers) that meets the primary endpoint requires the following: Admission to hospital or emergency room (until at least next calendar day) with symptoms presumed to be caused by myocardial ischemia with an accelerating tempo in the prior 48 h and/or prolonged (≥20 min) rest chest discomfort AND New high-risk ECG findings consistent with ischemia or infarction (or presumed new if no prior ECG available) as defined below: New or presumed new ST depression >0.5 mm in 2 contiguous leads or T wave inversion > 1 mm in leads with prominent R wave or R/S >1 in 2 contiguous leads, OR − new or presumed new ST elevation at the J point in >2 contiguous leads >0.2 mV in V2 or V3 in men or >0.15 mV in women in V2 or V3 or >0.1 mV in other leads. LBBB (*n*ew or presumed new) AND Definite contemporary evidence (defined below) of angiographically significant coronary disease as demonstrated by: Need for coronary revascularization procedure (PCI or CABG) excluding those performed to treat only restenosis lesion(s) at previous PCI site(s), OR − angiographic evidence of at least one significant (≥70%) epicardial coronary stenosis not due to restenosis at previous PCI site. Use of an adjudication committee: yes	UA: *n =* 97 Total: *n =* 1,955 UA/total: 5.0%

ARRIVE [45]	Patients were aged ≥55 years (men) or ≥60 years (women) and had a moderate cardiovascular risk (*n =* 12,546)	100 mg enteric-coated aspirin tablet once daily versus placebo	A composite outcome of time to first occurrence of CV death, Ml, UA, stroke, or transient ischemic attack	Intervention 4.3% versus control 4.5% (HR 0.96; 95% CI: 0.81 −1.13; *p =* 0.604)	The definition of UA was not further specified. Use of an adjudication committee: yes	UA: *n =* 40 Total: *n =* 550 UA/total: 7.2%

SPRINT [46]	Patients with SBP of ≥ 130 mm Hg and an increased CV risk, but without diabetes (***n*** = 9,361)	Randomization to a SBP target of <120 mm Hg versus <140 mm Hg	A composite of Ml, other ACS, stroke, heart failure, or death from CV causes	Intensive-treatment group 1.7% per year versus standard-treatment group 2.2% per year (HR with intensive treatment, 0.75; 95% CI: 0.64–0.89; *p* ***<*** 0.001)	Non-MI ACS was defined as hospitalization for evaluation and treatment of an accelerating or new symptom pattern consistent with coronary artery insufficiency without meeting the definition of Ml but requiring evaluation to rule-out Ml on clinical presentation. Non-MI ACS will also require objective findings of coronary ischemia. Non-MI ACS will be defined by one of 3 clinical presentations: (1) New cardiac symptoms and positive ECG findings with normal biomarkers not meeting criteria for Ml, OR (2) A changing symptom pattern and positive ECG findings with normal biomarkers not meeting criteria for Ml, OR (3) New or changing cardiac symptoms with normal biomarkers and ECGs but with further confirmatory evidence of CAD (e.g., 70% cross-sectional obstruction in at least one major coronary artery or branch on angiography at or near the time of admission, treatment with revascularization; prior documented CAD − prior CABG, PTCA, etc., positive exercise test at or near the time of admission, perfusion defect documented on stress scintigraphy or echo). Stress testing within approximately 1 month prior to admission or occurring during admission will be accepted as related to a particular hospital admission for possible non-MI ACS. Non-MI ACS requires an unscheduled admission that must have begun within 24 h of the most recent symptoms. Escalation of pharmacotherapy such as intravenous nitrates or increasing dosages of β-blockers, should be considered supportive but not diagnostic of non-MI ACS. Use of an adjudication committee: yes	Other ACS: *n =* 80 Total: *n =* 562 Other ACS/total: 14.2%

IMPROVE-IT [47]	Patients hospitalized for ACS within the preceding 10 days and elevated triglyceride levels (*n =* 18,144)	The combination of 40 mg simvastatin and 10 mg ezetimibe was compared versus 40 mg simvastatin and placebo	A composite of CV death, nonfatal Ml, UA requiring rehospitalization, coronary revascularization (≥30 days after randomization), or nonfatal stroke	Intervention 32.7% versus control 34.7% (HR 0.94; 95% CI: 0.89–0.99; *p* = 0.016)	An episode of ischemic discomfort consistent with UA (ischemic discomfort either at rest, of new onset, or in an accelerating pattern) lasting ≥10 min, which occurred before the subject presented to the hospital. The subject must then have been hospitalized (including a stay in the emergency department or observation unit of at least 12 h) and have had at least one of the following to meet the criteria for UA requiring rehospitalization: (a) Another episode of ischemic discomfort that occurred after arrival to the hospital and occurred at rest, lasting ≥ 10 min, was distinct from the episode that occurred outside of the hospital, and that was attributed to myocardial ischemia according to the treating physician. OR (b) New ST segment or new T-wave changes consistent with ischemia in two or more contiguous leads in association with an episode of ischemic discomfort. Note: If subjects were admitted with suspected UA, and subsequent testing revealed a noncardiac or nonischemic etiology, this would not be recorded as meeting this primary endpoint (or composite thereof). Use of an adjudication committee: yes	UA:n = 304 Total: *n =* 5,585 UA/total: 5.4%

JUPITER [48]	Apparently healthy men and women with LDL cholesterol levels of less than 130 mg/dL and high-sensitivity CRP of ≤2.0 mg/L (*n =* 17,802)	20 mg rosuvastatin daily versus placebo	A composite of Ml, stroke, arterial revascularization, hospitalization for UA, or death from CV causes	Intervention 0.8 per 100 person-years versus control 1.4 per 100 person-years (HR 0.56; 95% CI: 0.46–0.69; *p <* 0.001)	Adjudication on the basis of standardized criteria by an independent end-point committee unaware of the randomized treatment assignments. Further definition not specified. Use of an adjudication committee: yes	UA:n = 43 Total: *n =* 393 UA/total: 10.9%

PROVE-IT TIMI 22 [49]	Patients hospitalized for ACS within the preceding 10 days (*n =* 4,162)	40 mg of pravastatin daily versus 80 mg of atorvastatin daily	A composite of death from any cause, Ml, UA requiring rehospitalization and revascularization	Pravastatin 26.3% versus atorvastatin 22.4% (95% CI: 5–26%, *p* = 0.005)	UA was defined as ischemic discomfort at rest for at least 10 min prompting rehospitalization, combined with one of the following: ST-segment or T-wave changes, cardiac-marker elevations that were above the upper limit of normal but did not meet the criteria for Ml, or a second episode of ischemic chest discomfort lasting more than 10 min and that was distinct from the episode that had prompted hospitalization. Use of an adjudication committee: not specified	Not specified

RITA 3 [50]	Patients with NSTE-ACS (*n =* 1,810)	Conservative versus interventional therapy	A composite of death, nonfatal Ml, or refractory angina at 4 months; and a combined rate of death or non-fatal Ml at 1 year	Intervention 9.6% versus control 14.5% (risk ratio 0.66, 95% CI: 0.51–0.85, *p* = 0.001)	Refractory angina after randomization was diagnosed during the index hospital admission by the recurrence of suspected cardiac chest pain, with electrocardiographic evidence of ischemia, and provoking myocardial revascularization within 24 h of the onset of pain. After discharge, refractory angina was diagnosed if the patient was readmitted with an episode of cardiac chest pain associated with new electrocardiographic evidence of myocardial ischemia. Use of an adjudication committee: yes	UA:n = 124 Total: *n =* 219 UA/total: 56.6%

ACS, acute coronary syndrome; CI, confidence interval; CV, cardiovascular; HR, hazard ratio; LBBB, left bundle branch block; LVH, left ventricular hypertrophy; Ml, myocardial infarction; NSTE-ACS, non-ST-elevation acute coronary syndromes; SBP, systolic blood pressure; UA, unstable angina.

**Table 4:** Examples of contemporary clinical trials using revascularization as an endpoint

Trial name	Key inclusion criteria and sample size	Intervention versus control	Primary endpoint	Primary results	Definition of revascularization and use of an adjudication committee	Events, *n*
FAME–3 [52]	Patients with three-vessel CAD (*n* = 1,500)	FFR-guided PCI versus CABG	A composite of death from any cause, Ml, stroke, or repeat revascularization	FFR-guided PC1 10.6% versus CABG 6.9%. (HR 1.50; 95% CI: 1.10–2.20, *p* = 0.350 for noninferiority of FFR-guided PCI)	Urgent revascularization is defined as an unplanned hospitalization for an ACS with at least one of the following: electrocardiographic changes, biomarker elevation, or new perfusion/wall motion abnormalities to document ischemia and which results in revascularization during the hospitalization. Repeat revascularization is defined as any unplanned (elective or urgent) revascularization, whether PCI or CABG. Planned staged PCI procedures do not qualify. Use of an adjudication committee: yes	Revasc.: *n* = 74 Total: *n=* 131 Revasc./total: 56.4%

STEP [53]	Patients 60–80 years of age with hypertension (*n =* 9,624)	SBP target of 110–130 mm Hg (intensive treatment) versus 130–150 mm Hg (standard treatment)	A composite of stroke, ACS (acute Ml and UA), acute decompensated heart failure, coronary revascularization, atrial fibrillation, or death from cardiovascular causes	Intensive-treatment group 3.5% versus standard-treatment group 4.6%. (HR 0.74; 95% CI: 0.60–0.92; *p* = 0.007)	Coronary revascularization defined as PCI or CABG. Not further specified. Use of an adjudication committee: yes	Revasc.: *n* = 54 Total: *n =* 342 Revasc./total:15.8%

FLOWER–Ml [54]	Patients with STEMI and multivessel disease who had undergone successful PCI of the infarct-related artery (*n* = 1,163)	FFR versus angiography guided revascularization	A composite of death from any cause, nonfatal Ml, or unplanned hospitalization leading to urgent revascularization	FFR-guided 5.5% versus angiography-guided group 4.2% (HR 1.32; 95% CI: 0.78–2.23; *p* = 0.310)	Patient admitted to the hospital with persistent or increasing chest pain and the revascularization procedure (PCI or CABG surgery) performed during the same hospitalization. Use of an adjudication committee: yes	Revasc.: *n* = 26 Total: *n =* 56 Revasc./total: 46.4%

MR-INFORM [55]	Patients with typical angina and cardiovascular risk factors or a positive exercise treadmill test (*n =* 918)	Myocardial-perfusion cardiovascular MRI strategy versus invasive angiography and measurement of FFR strategy	A composite of death, nonfatal Ml, or target-vessel revascularization within 1 year	Cardiovascular-MRI group 3.6% versus the FFR group 3.7% (risk difference, −0.2 percentage points; 95% CI: −2.7 to 2.4)	Target vessel revascularization was defined as any repeat percutaneous intervention of the target lesion or bypass surgery of the target vessel performed for restenosis or other complication of the target lesion. The target lesion was defined as 5 mm within the revascularized segment. Use of an adjudication committee: yes	Revasc.: *n* = 10 Total: *n =* 31 Revasc./total: 32.3%

NOBLE [56]	Patients with left main CAD, stable angina, UA, or NSTEMI (*n* = 1,201)	PCI versus CABG for treatment of left main CAD	A composite of all-cause mortality, nonprocedural Ml, any repeat coronary revascularization, and stroke	PCI 29.0% versus CABG 19.0% (HR 1.48, 95% CI: 1.11 −1.96, *p =* 0.007)	Repeat revascularizations were categorized as target lesion revascularization, left main coronary artery target lesion revascularization, or de-novo lesion revascularization. Use of an adjudication committee: yes	Revasc.: *n* = 118 Total: *n =* 202 Revasc./total: 58.4%

FAME-2 [57]	Patients with stable CAD for whom PCI was being considered and in whom at least one stenosis was functionally significant (*n =* 1,220)	PCI versus medical therapy	A composite of death, Ml, or urgent revascularization	PCI 4.3% versus medical therapy 12.7% (HR with PCI 0.32; 95% CI: 0.19–0.53; *p <* 0.001)	Defined as unexpectedly hospitalization because of persisting or increasing complaints of chest pain (with or without ST-T changes, with or without elevated biomarkers) AND revascularization is performed within the same hospitalization. Use of an adjudication committee: yes	Revasc.: *n* = 56 Total: *n =* 75 Revasc./total: 74.7%

ACS, acute coronary syndromes; CABG, coronary artery bypass grafting; CAD, coronary artery disease; CI, confidence interval; CV, cardiovascular; FFR, fractional flow reserve; HR, hazard ratio; MRI, magnetic resonance imaging; NSTEMI, non-ST-elevation myocardial infarction; PCI, percutaneous coronary intervention; Revasc, revascularization; SBP, systolic blood pressure; STEMI, ST-elevation myocardial infarction; UA, unstable angina.
